# Erratum to: Genetic Biomarkers of Barrett’s Esophagus Susceptibility and Progression to Dysplasia and Cancer: A Systematic Review and Meta-Analysis

**DOI:** 10.1007/s10620-016-4192-4

**Published:** 2016-05-28

**Authors:** John M. Findlay, Mark R. Middleton, Ian Tomlinson

**Affiliations:** 1 Wellcome Trust Centre for Human Genetics, University of Oxford, Roosevelt Drive, Oxford, OX3 7BN UK; 2 Oxford OesophagoGastric Centre, Churchill Hospital, Oxford University Hospitals NHS Foundation Trust, Oxford, OX3 7LJ UK; 3 NIHR Oxford Biomedical Research Centre, The Joint Research Office, Churchill Hospital, Oxford, OX3 7LE UK; 4 Department of Oncology, Old Road Campus Research Building, University of Oxford, Roosevelt Drive, Oxford, OX3 7DQ UK

## Erratum to: Dig Dis Sci (2016) 61:25–38 DOI 10.1007/s10620-015-3884-5

The license for this article has been changed from “CC-BY-NC” to “CC-BY” at the author’s request.

